# The Effect of Expert Performance Microtiming on Listeners' Experience of Groove in Swing or Funk Music

**DOI:** 10.3389/fpsyg.2016.01487

**Published:** 2016-10-05

**Authors:** Olivier Senn, Lorenz Kilchenmann, Richard von Georgi, Claudia Bullerjahn

**Affiliations:** ^1^School of Music, Lucerne University of Applied Sciences and ArtsLucerne, Switzerland; ^2^Department of Social Sciences and Cultural Studies, Institute of Musicology and Music Education, Justus-Liebig-University GiessenGiessen, Germany; ^3^SRH Hochschule der populären KünsteBerlin, Germany

**Keywords:** microtiming, groove, entrainment, body movement, participatory discrepancies, funk, swing, musical expertise

## Abstract

This study tested the influence of expert performance microtiming on listeners' experience of groove. Two professional rhythm section performances (bass/drums) in swing and funk style were recorded, and the performances' original microtemporal deviations from a regular metronomic grid were scaled to several levels of magnitude. Music expert (*n* = 79) and non-expert (*n* = 81) listeners rated the groove qualities of stimuli using a newly developed questionnaire that measures three dimensions of the groove experience (*Entrainment, Enjoyment*, and the absence of *Irritation*). Findings show that music expert listeners were more sensitive to microtiming manipulations than non-experts. Across both expertise groups and for both styles, groove ratings were high for microtiming magnitudes equal or smaller than those originally performed and decreased for exaggerated microtiming magnitudes. In particular, both the fully quantized music and the music with the originally performed microtiming pattern were rated equally high on groove. This means that neither the claims of *PD theory* (that microtiming deviations are necessary for groove) nor the opposing *exactitude hypothesis* (that microtiming deviations are detrimental to groove) were supported by the data.

## 1. Introduction

Groove is a positive experience associated with rhythm and meter in music. Definitions of the concept differ in nuances, but there seems to be a consensus among musicians, music psychologists and music scholars that the groove experience consists in a person's inner urge to synchronize body movement with the beat of the music (Doffman, 2009; Keil, 2010; Janata et al., 2012; Davies et al., 2013; Frühauf et al., 2013; Madison and Sioros, 2014; Sioros et al., 2014; Witek et al., 2014). The groove experience is further considered to be an enjoyable experience, and it is characterized by an impression of effortlessness and flow (Berliner, 1994, p. 389). According to Pfleiderer (2006), the groove concept is mostly used with respect to North American popular music (jazz, funk, R&B, soul and others). In a more general sense, groove has been understood as a transcultural phenomenon independent of the concept's roots in North American genres (Klingmann, 2010), and it has been used to analyze the effect of rhythmic music from different cultural backgrounds (Madison et al., 2011).

The use of music in connection with body movement (dance, work, sports, military drill) is ubiquitous. Accordingly, the groove phenomenon has received considerable scholarly attention in recent years, also due to a growing interest in embodiment and musical entrainment (starting with Clayton et al., 2005). Music must satisfy two preconditions in order to trigger entrainment: firstly, listeners must be able to recognize the music's metric and rhythmic regularities (Large and Jones, 1999; Merker, 2014). Secondly, these regularities must stimulate some kind of resonance in the listeners' minds and/or bodies (Noorden and Moelants, 1999).

A major research focus lies on studying those properties of music that enhance or diminish groove. One popular theory (*PD theory*) claims that microtemporal patterns arising in music performance—small timing deviations from strict metronomic time, often within a range of ±50 ms—are crucial for the creation of groove. This theory is based on Charles Keil's concept of *Participatory Discrepancies* or *PD*s (Keil, 1987, 1995, 2010). The theory is substantially based on the expertise of professional musicians, and it appears to have a considerable number of followers within this population. This is confirmed in the scholarly literature (Berliner, 1994; Monson, 1996; Greenwald, 2002; Doffman, 2008) and in magazines on jazz or popular music (Hoinkis, 2013).

Since 2010, several empirical studies have tested the validity of *PD theory*. Butterfield (2010) found that listeners failed to consistently detect PD-sized microtiming deviations (up to a magnitude of 30 ms), and he concluded that PDs were unlikely to be relevant for groove. Two studies (Madison et al., 2011; Madison and Sioros, 2014) found no correlations between the magnitude of microtiming deviations and groove ratings; instead they reported correlations between groove and other musical properties like event density, beat salience, or syncopation (the relevance of syncopation for groove was further elaborated by Sioros et al., 2014; Witek et al., 2014). Frühauf et al. (2013) and Davies et al. (2013) reported that the groove phenomenon is indeed related to microtiming, albeit negatively: large microtemporal deviations were associated with low groove ratings and, vice-versa, the completely quantized stimuli (i.e., the stimuli with strict metronomic timing) obtained the highest groove ratings.

Taken together, the previous studies found only little evidence for *PD theory*'s assumption that some level of microtemporal deviations contributes positively to the groove experience. Merker (2014) argued that the claim of *PD theory* was counter-intuitive, since microtiming deviations rather obscured metric and rhythmic regularities instead of clarifying them. This line of thinking may be summarized under the heading *exactitude hypothesis*: it claims that groove is positively associated with timing precision. Under this hypothesis, perfectly quantized music triggers a more intense groove experience than music with timing deviations.

Kilchenmann and Senn (2015) addressed the claims of *PD theory* and the *exactitude hypothesis* by measuring the actual bodily entrainment response to microtiming manipulations in swing and funk music examples, using video-based motion tracking technology. The data suggest that the timing manipulations had significant effects on the behavior of music expert listeners, while no effects were measured in non-expert listeners. The results conflicted with the *exactitude hypothesis* insofar as the fully quantized stimuli were not associated with strong entrainment in experts. Instead, stimuli with tight but non-zero microtiming triggered the largest entrainment reaction. Furthermore, entrainment behavior in experts was not related to musical genre (swing, funk). A surprising result was that the stimuli with loosest timing triggered strong entrainment in music experts.

These results cannot directly be compared to earlier findings given the different methodological approaches: In Kilchenmann and Senn (2015), data on bodily behavior were used as a measure of entrainment, whereas the earlier studies used questionnaires to assess listeners' groove experience. The present paper closes this gap: It reports results from questionnaire data that were collected during the same experiment that created the movement data for Kilchenmann and Senn (2015). In so doing it triangulates the previously published findings and offers new insights on their scope and interpretation.

A major concern for the assessment of groove through questionnaires is what dimensions of the experience should be measured and what questions or statements should be used to measure them. Janata et al. (2012) in their second experiment asked participants to rate 148 commercially available popular music recordings answering the question how much the music “grooved.” The participants gave feedback using a slider; this resulted in ratings on a quasi-continuous Likert scale. This direct approach (asking explicitly about groove) might cause genre bias: raters might be influenced by the fact that some musical styles are traditionally associated with groove, while others are not.

Madison et al. (2011) operationalized groove in terms of entrainment: groove in music “evokes the sensation of wanting to move some part of the body.” They avoided genre bias by not using the groove concept directly in their questionnaire. On an 11-point Likert scale participants rated the extent to which music was experienced as being “motion generating.” They also collected information on familiarity and overall music quality, but the “motion generating” item was used as the primary indicator for groove. The same item was also used by Davies et al. (2013) and Sioros et al. (2014) as main measure of groove.

In Witek et al. (2014) participants rated how much the rhythm made them want to move and how much pleasure they experienced while listening. The ratings were collected using 5-point Likert scales. Frühauf et al. (2013) considered a multitude of aspects that seem to be important for the groove phenomenon. Using quasi-continuous 101-point Likert scales they assessed the execution of timing, the performance in general, the felt entrainment/animation, whether listeners liked the music, and its overall aesthetic quality. The overall groove rating was computed as a composite measure of the five dimensions.

In this study we developed a new psychometric tool, the *Emotional Assessment of Groove* (*EAG*) questionnaire, to measure the intensity of listeners' groove experience. The questionnaire captures three basic dimensions: listeners' felt *Entrainment, Enjoyment*, and the music's naturalness and flow, assessed by inversely measuring the degree of *Irritation* experienced by the listeners.

The goal of this study was to clarify the role of real-world performance microtiming in the subjective groove experience of expert and non-expert listeners by systematically manipulating the magnitude of the microtiming deviations in short recorded funk and swing clips. In line with Kilchenmann and Senn (2015) and *PD theory* we hypothesized that the music examples with the original microtiming patterns (as played by the musicians) would receive higher mean groove ratings than music examples with manipulated timing. Specifically, we hypothesized that the groove ratings would be lower the more the timing differed from the originally performed timing.

The relevance of listeners' musical expertise for the groove effect of microtiming is, as yet, unclear: Davies et al. (2013) and Kilchenmann and Senn (2015) showed that experts reacted more strongly to microtiming manipulations than non-experts, but Frühauf et al. (2013) did not observe such an effect. We hypothesized that expert listeners would be more sensitive to timing manipulations than non-experts and thus give more differentiated groove ratings. Finally, based on the notion that funk is the prototypical groove-related music genre (Danielsen, 2006; Southgate, 2011), we hypothesized that the funk clips would receive higher groove ratings than the swing clips. By discussing the movement data of Kilchenmann and Senn (2015) along with the subjective questionnaire data of the present study, we also hope to shed light on the relationship between listeners' observable behavior and their subjective experience.

## 2. Materials and methods

### 2.1. Stimuli

The experimental stimuli were derived from recorded performances by two professional and internationally renowned musicians, bassist Wolfgang Zwiauer and drummer Dominik Burkhalter. Two recordings were made during a studio jam session for the specific use in this experiment. In one recording, the musicians played an eight-bar funk pattern at 100 bpm on drums and electric bass during several minutes. In another recording, they played a twelve-bar swing pattern at 150 bpm on drums and acoustic bass guitar. The musicians extemporated the music after agreeing on some basic features.

During the performance, the musicians heard a metronome click over headphones as a common beat reference; the metronome click was recorded to a separate track. To hear a metronome click while playing is common practice in studio work, and the musicians confirmed being comfortable with it. After the recording session, the musicians indicated segments from each recording that, in their opinion, had the best groove. From these segments, the experimenters chose one iteration of each pattern. As a result, we retained 20 s of music for each style that would serve as basis for the timing manipulations and for creating the experimental stimuli. The musicians agreed with this choice.

**Figure 1:**
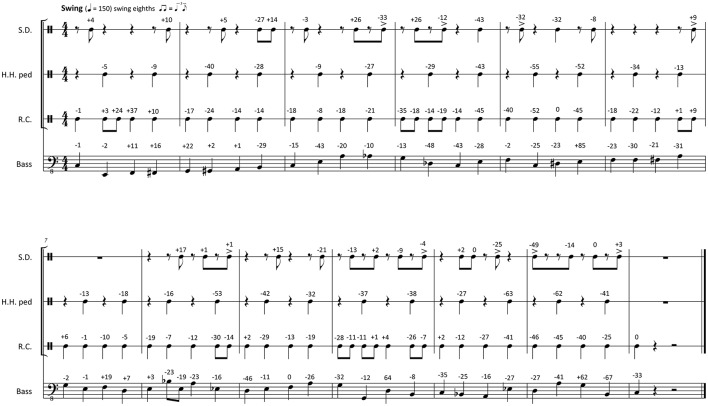
**Transcription of the swing stimuli with original PDs notated in milliseconds above each note (negative numbers, ahead compared to metronomic time grid; positive numbers, late compared to metronomic time grid; S.D., snare drum; H.H. ped, foot-operated hi-hat cymbal; R.C., ride cymbal)**.

**Figure 2:**
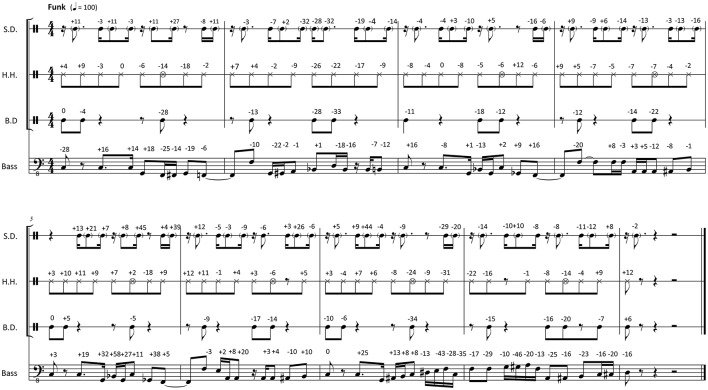
**Transcription of the funk stimuli with original PDs notated in milliseconds above each note (negative numbers, ahead compared to metronomic time grid; positive numbers, late compared to metronomic time grid; S.D., snare drum; H.H., hand-operated hi-hat cymbal; B.D., bass drum)**.

Figures 1, 2 show transcriptions of the musical passages used for the experiment. The transcriptions have been created by the researchers after the recording session on the basis of the recorded music. Subsequently, the musicians checked whether the transcriptions were accurate and idiomatic. From the click track and the transcribed rhythm, a metronomic grid was derived, which defined regular, quantized onset times for the events on each metrical position. Then the timing differences between these quantized times and the performed note onset times were calculated. These microtiming deviations from the metronomic grid were defined to represent the *Participatory Discrepancies* (*PD*s) of the performance with respect to timing. The *PD*s are given in the transcriptions as a numeric value above each note.

In a second step, 12 versions were produced for each of the two original recordings (swing and funk) by scaling the microtiming deviations with respect to the metronomic grid. The manipulations were governed by two variables: the *Direction* and the Δ*-Magnitude* of the scaling.

The *Direction* variable had two levels, Reduction and Expansion. For Reduction the original deviations were downscaled (in musicians' parlance: the timing gets “tighter”), for Expansion they were upscaled (the timing gets “looser”). The Δ*-Magnitude* variable determined by which percentage the deviations would be down- or upscaled; it had six levels (0, 20, 40, 60, 80, or 100%). Table 1 shows as an example, how a timing deviation of −15 ms (15 ms early compared to the metronomic grid) would have been treated across the twelve stimuli. At a Δ*-Magnitude* of 0%, the onset's deviation from the metronomic grid is exactly as in the original recorded performance for both *Direction* levels (−15 ms). At 100% Reduction, the deviation shrinks to 0 ms, so that the onset is exactly on the metronomic grid. At 100% Expansion, the deviation doubles (−30 ms).

**Table 1:** **Timing manipulations for a note onset that is 15 ms early in the original performance, compared to the metronomic grid**.

***Δ-Magnitude***	**0%**	**20%**	**40%**	**60%**	**80%**	**100%**
**Direction**						
Reduction	−15	−12	−9	−6	−3	0
Expansion	−15	−18	−21	−24	−27	−30

Deviations from the grid are given in milliseconds.

Each individual event onset was down- or upscaled according to this rule, based on its original timing deviation from the metronomic grid. As a result the 0% Reduction and Expansion versions were identical and their timing was exactly as played by the musicians. In the Reduction series, the timing became tighter with higher levels of Δ*-Magnitude*; at the 100% Reduction level, the music was perfectly quantized. In the Expansion series, the timing became looser with higher levels of of Δ*-Magnitude*. At the 100% Expansion level, deviations were doubled in magnitude.

With 12 stimuli in either style a total of 24 stimuli was prepared for the experiment. For further details about the preparation of the recordings and the creation of the stimuli, please refer to Kilchenmann and Senn (2015). The stimuli can be downloaded from the Supplementary Material Section of this earlier study.^1^

### 2.2. Psychometric measures

#### 2.2.1. Groove experience

The twenty-item *Emotional Assessment of Groove* (*EAG*) questionnaire was developed by the authors during a workshop in Lucerne in 2012. The aim of the questionnaire was to obtain listeners' feedback on three aspects that have consistently been associated with the groove experience: (1) participants' urge to move their bodies while listening to music; (2) their feeling of enjoyment, and (3) the effortlessness and flow of the music. The last aspect was inversely conceived as a degree of irritation.

Answers were collected on five-point Likert scales (for scale construction, see McIver and Carmines, 1981; Gliem and Gliem, 2003; Nunnally and Bernstein, 2010). The twenty-item questionnaire can be inspected in the Supplementary Material Section of this article.

The questionnaire was validated at Justus-Liebig-University Giessen with 90 students who listened to stimuli unrelated to the experiment. Factor analysis revealed a three-factor structure, as intended by the authors: 9 items loaded on a first factor related to listeners' feeling of *Entrainment* (Cronbach's α = 0.92). Five items loaded on a factor related to their interest or *Enjoyment* of the music (α = 0.88). And finally 5 items loaded on a factor concerning the listeners' impression of unnaturalness of the stimuli or their feeling of *Irritation* (α = 0.97).

The actual experiment was carried out in Lucerne, and the experimental data confirmed the three-factor structure. Thirteen items had factor loadings of ≥|0.5| on one of the three factors, and they were sufficient to create reliable scales; the additional 7 items did not augment reliability. Hence, only these 13 items were used for the present analyses. Items and factor loadings are presented in Table 2. The questionnaire was presented to the participants in German. The English translations of the items have been added in the table for reader convenience only. They have neither been validated, nor have they been used in the experiment.

**Table 2:** **Factor analysis of experimental ***EAG*** questionnaire data**.

**No**.	**Item**	**Entrainment**	**Enjoyment**	**Irritation**
16	Ich hatte das Gefühl, dass sich mein Kopf zum Rhythmus mitbewegt.	0.89		
	*I had the impression that my head moved with the rhythm.*			
13	Ich hatte das Gefühl, dass ich mit meinem Fuss gerne mitklopfen würde.	0.88		
	*I felt like tapping my foot with the music.*			
2	Das Beispiel animierte mich zum Mitwippen.	0.82		
	*The music stimulated me to bop along.*			
5	Das Beispiel animierte mich zum Mitklatschen oder -schnippen.	0.63		
	*The music stimulated me to clap along or click my fingers.*			
9	Ich empfand das Beispiel als frisch.		0.84	
	*To my impression the music sounded fresh.*			
7	Ich empfand das Beispiel als anregend.		0.82	
	*To my impression the music was stimulating.*			
12	Das Beispiel war für mich sehr kraftvoll.		0.81	
	*In my opinion the music had a lot of power.*			
14	Ich empfand das Beispiel als eher langweilig.		−0.64	
	*To my impression the music was rather boring.*			
15	Bei diesem Beispiel hätte mich interessiert, wie es weitergeht.		0.58	
	*I would have been interested to know how the music continues.*			
11	Das Beispiel hinterliess den Eindruck einer gewissen Holprigkeit,			0.92
	die mir eher unangenehm war.			
	*The music made impression of unevenness, which was rather unpleasant.*			
17	Irgendetwas war mit dem Beispiel nicht in Ordnung und ich hatte			0.90
	ein merkwürdiges Gefühl.			
	*Something in the music was not in order, and I had a weird feeling.*			
3	Ich hatte das Gefühl, dass irgendetwas störend wirkt.			0.89
	*I had the impression that something in the music bothered me.*			
8	Irgendwie wirkte das Beispiel bremsend und/oder merkwürdig auf mich.			0.83
	*The music dragged and/or made a strange impression on me.*			
	Reliability (Cronbach's Alpha)	0.89	0.88	0.94

Factor loadings are only given if their absolute value is ≥ 0.50.

Cronbach's α was 0.89 for the four *Entrainment* items, 0.88 for the five *Enjoyment* items, and 0.94 for the four *Irritation* items. Overall, the reliability of the scales is good according to the standards defined by Nunnally and Bernstein (2010). In concordance with previous studies on groove, we expected participants to express a strong groove experience by high *Entrainment* ratings, high *Enjoyment* ratings, and low *Irritation* ratings.

#### 2.2.2. Affective reactions

In addition to the *EAG*, the *Self Assessment Manikin* (*SAM*) questionnaire was used to measure participants' affective reactions to each listening experience (Bradley and Lang, 1994; Backs et al., 2005). This tried-and-tested pictorial questionnaire allows for subjects to express their affective state in three dimensions: *Valence* (happy/unhappy), *Arousal* (quiet/excited), and *Dominance* (powerful/powerless). It has successfully been used to measure affective reactions to music in the past (e.g., Gomez and Danuser, 2007).

#### 2.2.3. Additional measures: general affective disposition, personality

Listeners' reactions to music can be expected to depend on their personality (Payne, 1967; Rawlings and Ciancarelli, 1997; Delsing et al., 2008). Participants self-assessed their affective disposition filling the German trait version of the 20-item *Positive and Negative Affect Schedule* or *PANAS-d* (Watson et al., 1988; Krohne et al., 1996; Crawford and Henry, 2004). They were asked to assess their affective state “in general” (while other versions of the test address shorter time frames, like “today” or “this week”).

Personality traits were measured using the German version of the well-established *NEO Five Factor Inventory*, a 60-item questionnaire that measures five broad personality traits from a subjective perspective: *Openness to experience, Conscientiousness, Extraversion, Agreeableness*, and *Neuroticism* (McCrae and Costa, 1987, 2003; Borkenau and Ostendorf, 2008).

### 2.3. Participants, setup, and procedure

One hundred sixty participants were recruited at the Lucerne University of Applied Sciences and Arts and at Lucerne University. Seventy nine participants were considered to be music experts: they were enrolled in a program to become professional music performers or music teachers (Bachelor/Master of Arts in Music or Music Pedagogy). The experts had 13 years of median experience practicing a musical instrument (*IQR* = 6). The remaining 81 participants were considered to be musical non-experts. They were enrolled in other, non music-related programs and had 5 years of median experience practicing a musical instrument (*IQR* = 8.25). Overall, there were 82 female and 78 male participants; their mean age was 24.4 years (*SD* = 4.3). All participants were fluent German speakers.

The experiment was carried out in a quiet university office. Participants took the listening test one person at a time. The technical setup of the experiment is given in detail in Kilchenmann and Senn (2015). During the pretest phase, participants read an information leaflet. They were informed that the experiment was about music perception; no reference to microtiming, groove, or musical entrainment was given. They were informed that they could abort the experiment at any time. The pretest phase included a gap detection test to assess participants' auditory timing discrimination. The participants' mean auditory time resolution was 1.7 ms (*SD* = 0.65, *min* = 1, *max* = 4). No participant was excluded from the experiment because of the gap detection test result. Participants listened to three test stimuli, practiced filling the *SAM* and *EAG* questionnaires presented on the screen, and adjusted playback loudness to a comfortable level. Finally, they could ask questions if anything about the procedure was unclear.

After the trial runs, the experimenter left the room, and the participant was guided through the experiment by on-screen instructions. Each participant was randomly assigned to one *Style* (Swing, Funk). For each *Style*, 12 stimuli were presented, grouped in two *Direction* series (Expansion, Reduction), each consisting of six stimuli with different Δ*-Magnitude*. The presentation of the two series and of the stimuli within the two series was randomly ordered for each participant. Participants triggered the stimuli themselves and filled the *EAG* and *SAM* questionnaires after each stimulus. This allowed us to capture their affective state immediately after the listening experience. After completing the first *Direction* series, they filled the *PANAS-d* questionnaire, and after the second series the *NEO-ffi* questionnaire. The Ethics Commission of the Canton of Lucerne approved of the design and the procedure of the experiment.

### 2.4. Statistical design

Mixed-design analyses of variance were performed using *R* (version 3.0.2). There were six dependent variables: *Valence, Arousal*, and *Dominance* from the *SAM* questionnaire, and *Entrainment, Enjoyment*, and *Irritation* from the *EAG* questionnaire. Out of the six dependent variables, the EAG's *Irritation* scale was positively skewed (γ_1_ = 0.74), while all others were approximately normally distributed. In spite of the non-normality of the distribution, the *Irritation* data were kept in the analyses: The limited range of the Likert-type scale prevents extreme outliers; hence the effects of non-normality can be estimated to be mild.

In the original design, there were four independent variables, two of them between-subjects: *Style* (Funk, Swing) and *Expertise* (Expert, Non-Expert). Two further variables were within-subjects: *Direction* (Reduction, Expansion) and Δ-*Magnitude* (0, 20, 40, 60, 80, 100%) encoded the timing manipulations.

During the peer review process for this paper it became clear that the parametrization of the timing manipulations using *Direction* and Δ*-Magnitude* was problematic. In particular, the Δ*-Magnitude* variable turned out to be poorly specified: its levels pool responses to stimuli with diverging timing patterns. This led to heteroscedasticity among the levels of Δ*-Magnitude*; and the main effect of the variable was not interpretable. In order to solve this problem, the timing manipulations were newly encoded using a variable called *Signed-*Δ*-Magnitude*. Table 3 shows the allocation rule that governs how combinations of Δ*-Magnitude* and *Direction* levels were projected onto the levels of *Signed-*Δ*-Magnitude*. The combinations from the Reduction series are negatively signed and those from the Expansion series have positive signs. The numeric values from Δ*-Magnitude* have not changed, except for the sign. The two Δ*-Magnitude*/*Direction* combinations referring to the stimuli with the originally performed timing (0% Reduction, 0% Expansion) have both been preserved as separate levels of *Signed-*Δ*-Magnitude* (−0%, +0%) in order to keep group sizes balanced; so, *Signed-*Δ*-Magnitude* has twelve levels. In summary, the re-encoding maps the timing manipulations from the two-parameter Δ*-Magnitude* / *Direction* setup onto a single *Signed-*Δ*-Magnitude* variable that orders the data according to the size of the PDs in the stimuli.

**Table 3:** **Re-encoding of the timing manipulations**.

**Δ-Magnitude (%)**	**Direction**	**→**	**Signed-Δ-magnitude (%)**
100	Reduction	→	−100
80	Reduction	→	−80
60	Reduction	→	−60
40	Reduction	→	−40
20	Reduction	→	−20
0	Reduction	→	−0
0	Expansion	→	+0
20	Expansion	→	+20
40	Expansion	→	+40
60	Expansion	→	+60
80	Expansion	→	+80
100	Expansion	→	+100

After re-encoding the timing manipulations, the data was analyzed using three independent variables: *Style, Expertise* (both between subjects), and *Signed-*Δ*-Magnitude* (within subjects). The three-way mixed-model ANOVAs tested for effects on all dependent variables: *Valence, Arousal, Dominance, Entrainment, Enjoyment*, and *Irritation*. The overall significance level was set to α = 0.05. Šidàk correction (Šidàk, 1967; Huberty and Morris, 1989) was applied to control the familywise Type I error rate. This resulted in a significance level of α = 0.0085 for the single ANOVAs.

For one participant (Non-Expert, Swing), measurements were incomplete due to technical problems; this participant's data were excluded from analysis. Hence, the following results are based on a sample of 159 participants.

## 3. Results

The results of the analyses of variance are reported in Table 4. The timing manipulations of *Signed-*Δ*-Magnitude* had a highly significant effect on *Valence, Entrainment, Enjoyment*, and *Irritation*. The main effect of *Signed-*Δ*-Magnitude* on *Dominance* was only near-significant due to the Šidàk correction.

**Table 4:** **Omnibus significance tests (ANOVA)**.

**Source**	**DV**	**SS**	***df***	**MSS**	***F***	***p***
Style	Valence	15.3	1	15.275	0.995	0.320
	Arousal	10.7	1	10.704	0.739	0.391
	Dominance	0.8	1	0.758	0.078	0.781
	Entrainment	0.4	1	0.355	0.036	0.850
	Enjoyment	2.7	1	2.683	0.459	0.499
	Irritation	19.9	1	19.850	3.403	0.067
Expertise	Valence	13.4	1	13.384	0.872	0.352
	Arousal	0.0	1	0.035	0.002	0.961
	Dominance	19.4	1	19.378	1.984	0.161
	Entrainment	7.1	1	7.136	0.726	0.396
	Enjoyment	10.3	1	10.332	1.766	0.186
	Irritation	0.2	1	0.163	0.028	0.867
Style × expertise	Valence	46.9	1	46.919	3.057	0.082
	Arousal	0.3	1	0.259	0.018	0.894
	Dominance	0.0	1	0.047	0.005	0.945
	Entrainment	0.0	1	0.001	0.001	0.992
	Enjoyment	2.6	1	2.546	0.435	0.511
	Irritation	0.0	1	0.006	0.001	0.976
Signed-Δ-magnitude	Valence	249.7	11	22.696	10.549	< 0.001^*^
	Arousal	32.8	11	2.985	1.528	0.130
	Dominance	42.2	11	3.835	2.151	0.019
	Entrainment	61.9	11	5.626	11.141	< 0.001^*^
	Enjoyment	39.2	11	3.566	6.523	< 0.001^*^
	Irritation	229.2	11	20.837	24.470	< 0.001^*^
Signed-Δ-magnitude × style	Valence	23.1	11	2.104	0.978	0.464
	Arousal	44.3	11	4.029	2.063	0.029
	Dominance	14.9	11	1.353	0.762	0.678
	Entrainment	5.7	11	0.522	1.034	0.413
	Enjoyment	5.6	11	0.504	0.922	0.518
	Irritation	10.2	11	0.923	1.084	0.383
Signed-Δ-magnitude × expertise	Valence	26.9	11	2.444	1.136	0.333
	Arousal	14.7	11	1.336	0.684	0.755
	Dominance	19.9	11	1.807	1.019	0.427
	Entrainment	11.4	11	1.039	2.057	0.030
	Enjoyment	12.2	11	1.106	2.023	0.030
	Irritation	28.8	11	2.619	3.076	0.001^*^
Signed-Δ-magnitude × style × expertise	Valence	26.4	11	2.397	1.114	0.349
	Arousal	23.6	11	2.146	1.099	0.360
	Dominance	15.1	11	1.372	0.774	0.667
	Entrainment	3.8	11	0.342	0.677	0.762
	Enjoyment	4.6	11	0.415	0.759	0.682
	Irritation	10.5	11	0.951	1.117	0.348

DV, dependent variable; SS, sum of squares; df, degrees of freedom; MSS, mean sum of squares; F, F-statistic; p, significance probability (

* p ≤ 0.0085). Greenhouse-Geisser correction was applied to all effects involving Signed-Δ-Magnitude.

A significant *Signed-*Δ*-Magnitude*×*Expertise* interaction effect was measured on the *Irritation* scale. The *Signed-*Δ*-Magnitude*×*Expertise* interaction effects on *Entrainment* and *Enjoyment* were near-significant. *Style* and all interactions involving *Style* did not have a significant effect on any of the dependent variables.

### 3.1. *Signed*-Δ-*Magnitude* main effect

**Figure 3:**
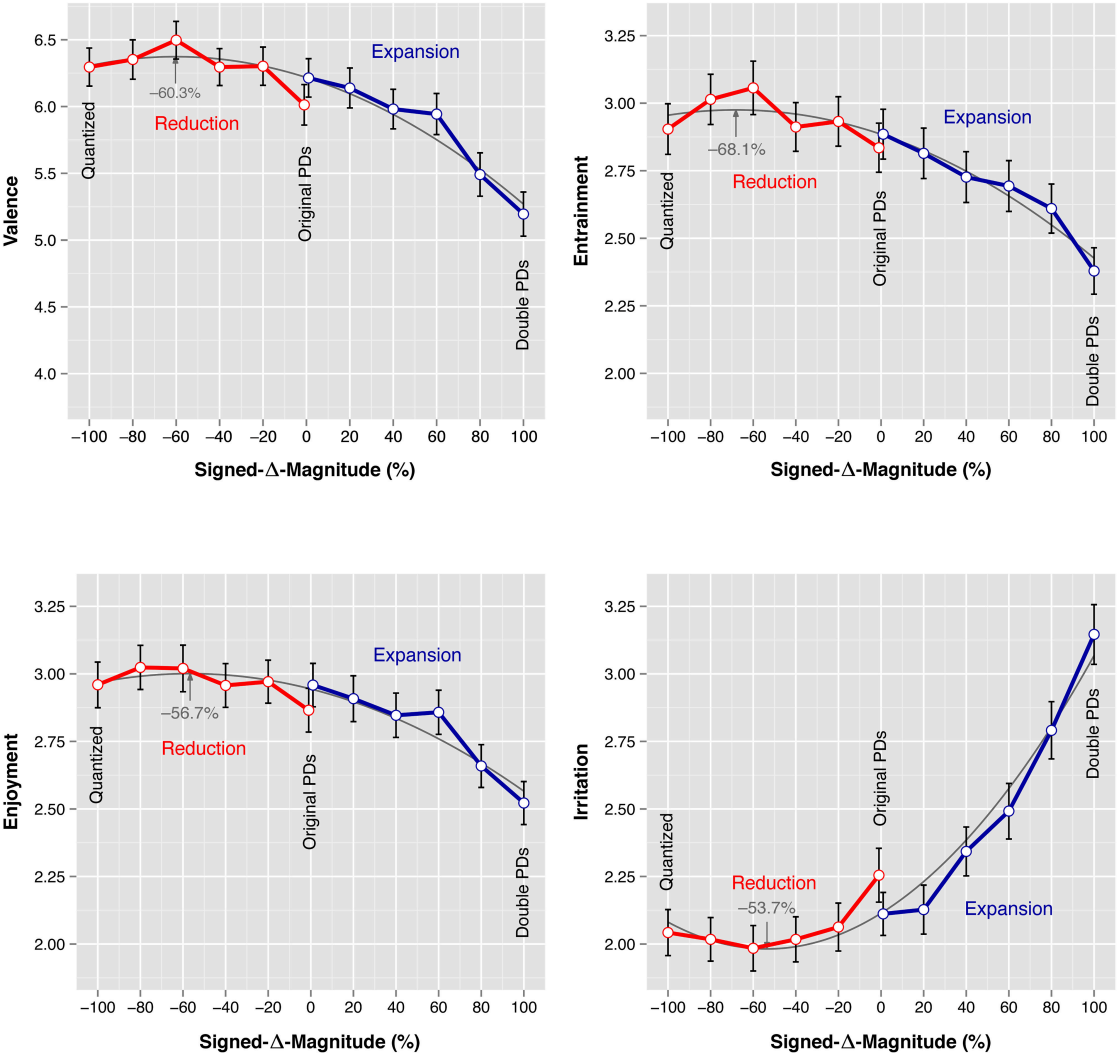
**Mean ***Valence*** (SAM), ***Entrainment***, ***Enjoyment***, and ***Irritation*** (***EAG***) ratings for each level of ***Signed***-Δ-***Magnitude*****. Quadratic polynomial lines of best fit are given in the background, extrema are indicated by arrows. Error bars represent the standard error of the mean.

Figure 3 plots the mean *Valence, Entrainment, Enjoyment*, and *Irritation* responses for all levels of *Signed-*Δ*-Magnitude*. For convenience, the two original *Direction* series are marked with red (Reduction) and blue lines (Expansion). We observe similar patterns across the mean *Valence, Entrainment*, and *Enjoyment* ratings. The ratings were high for negatively signed levels of *Signed-*Δ*-Magnitude*; and they decreased for positively *Signed-*Δ*-Magnitude* levels. The mean *Irritation* ratings mirror this pattern: listeners were little irritated while listening to the stimuli of the negatively signed *Signed-*Δ*-Magnitude* levels, but *Irritation* increased for higher positively signed levels of *Signed-*Δ*-Magnitude*.

*Post-hoc* pairwise Tukey HSD tests were carried out to pinpoint effects between the twelve *Signed-*Δ*-Magnitude* levels (Table 5; for compactness only comparisons with significant effects on any of the dependent variables were listed). The pairwise comparisons confirm the observations obtained from Figure 3: no pairwise effects were measured between any of the lower levels of *Signed-*Δ*-Magnitude* from −100% up to and including +20%. However, the higher levels of *Signed-*Δ*-Magnitude* (+40, +60, +80, and +100%) were rated low on groove in comparison to at least one of the other levels of *Signed-*Δ*-Magnitude*. *Irritation* registered these effects most markedly; the largest effect was measured between the −60% and +100% levels of *Irritation* (*p* < 0.001, *d* = 0.966). The −100% level (quantized stimuli) and the −0% and +0% (originally performed timing) levels obtained high groove ratings that were not significantly different from each other on any of the scales.

**Table 5:** *****Post-hoc*** pairwise comparisons (Tukey HSD) of mean ratings for levels of ***Signed-***Δ***-Magnitude*****.

**Signed-**Δ**-magnitude**			**Valence**		**Entrainment**		**Enjoyment**		**Irritation**	
			***p***	***d***	***p***	***d***	***p***	***d***	***p***	***d***
−100%	⇄	+60%	0.594	–	0.260	–	0.987	–	0.001	0.388
		+80%	< 0.001	0.434	0.013	0.261	0.016	0.301	< 0.001	0.635
		+100%	< 0.001	0.587	< 0.001	0.479	< 0.001	0.437	< 0.001	0.912
−80%	⇄	+40%	0.510	–	0.017	0.252	0.598	–	0.078	–
		+60%	0.351	–	0.004	0.281	0.695	–	< 0.001	0.419
		+80%	< 0.001	0.457	< 0.001	0.360	0.001	0.374	< 0.001	0.670
		+100%	< 0.001	0.608	< 0.001	0.581	< 0.001	0.512	< 0.001	0.951
−60%	⇄	+40%	0.076	–	0.002	0.280	0.631	–	0.029	0.337
		+60%	0.038	0.310	< 0.001	0.309	0.726	–	< 0.001	0.442
		+80%	< 0.001	0.545	< 0.001	0.385	0.001	0.358	< 0.001	0.689
		+100%	< 0.001	0.699	< 0.001	0.600	< 0.001	0.492	< 0.001	0.966
−40%	⇄	+60%	0.594	–	0.210	–	0.989	–	< 0.001	0.414
		+80%	< 0.001	0.441	0.009	0.274	0.018	0.306	< 0.001	0.662
		+100%	< 0.001	0.596	< 0.001	0.497	< 0.001	0.445	< 0.001	0.940
−20%	⇄	+60%	0.566	–	0.112	–	0.970	–	0.002	0.365
		+80%	< 0.001	0.436	0.003	0.290	0.010	0.323	< 0.001	0.608
		+100%	< 0.001	0.589	< 0.001	0.512	< 0.001	0.463	< 0.001	0.882
−0%	⇄	+80%	0.068	–	0.175	–	0.352	–	< 0.001	0.425
		+100%	< 0.001	0.424	< 0.001	0.423	0.002	0.352	< 0.001	0.689
+0%	⇄	+60%	0.893	–	0.406	–	0.988	–	0.014	0.336
		+80%	< 0.001	0.388	0.029	0.246	0.017	0.309	< 0.001	0.589
		+100%	< 0.001	0.542	< 0.001	0.465	< 0.001	0.449	< 0.001	0.873
+20%	⇄	+60%	0.990	–	0.937	–	0.999	–	0.024	0.306
		+80%	0.005	0.342	0.305	–	0.110	–	< 0.001	0.549
		+100%	< 0.001	0.493	< 0.001	0.398	< 0.001	0.386	< 0.001	0.821
+40%	⇄	+80%	0.115	–	0.952	–	0.509	–	0.001	0.371
		+100%	< 0.001	0.412	< 0.001	0.316	0.006	0.330	< 0.001	0.649
+60%	⇄	+100%	< 0.001	0.386	0.005	0.287	0.003	0.342	< 0.001	0.498
+80%	⇄	+100%	0.821	–	0.144	–	0.890	–	0.032	0.267

p, significance level; d, Cohen's d. Comparisons without significant effects on any of the dependent variables are omitted.

The plots of Figure 3 suggest fairly consistent curvilinear dose-response relationships between *Signed-*Δ*-Magnitude* and the mean ratings. In order to illustrate these relationships, quadratic regression models were fitted to the 1908 data points for each dependent variable. Table 6 shows the estimated model coefficients for *Valence, Entrainment, Enjoyment*, and *Irritation*. In each case, the quadratic model fit was significantly better than the best alternative linear model. The quadratic models' lines of best fit are printed in the background of the Figure 3 plots, and they summarize the data reasonably well.

**Table 6:** *****Post-hoc*** quadratic polynomial regression models for the relationship between ***Signed-***Δ***-Magnitude*** (predictor) and four responses (***Valence, Entrainment, Enjoyment***, and ***Irritation***)**.

**DV**	**Coefficient**	**Estimate**	**SE**	***df***	***t***	***p***
Valence	Intercept	6.217 × 10^−0^	5.938 × 10^−2^	1905	104.697	< 0.001
	Linear	−5.183 × 10^−3^	6.830 × 10^−4^	1905	−7.589	< 0.001
	Quadratic	−4.295 × 10^−5^	1.162 × 10^−5^	1905	−3.695	< 0.001
Entrainment	Intercept	2.885 × 10^−0^	3.703 × 10^−2^	1905	77.912	< 0.001
	Linear	−2.646 × 10^−3^	4.258 × 10^−4^	1905	−6.213	< 0.001
	Quadratic	−1.942 × 10^−5^	7.246 × 10^−6^	1905	−2.680	0.007
Enjoyment	Intercept	2.944 × 10^−0^	3.266 × 10^−2^	1905	90.151	< 0.001
	Linear	−2.009 × 10^−3^	3.756 × 10^−4^	1905	−5.348	< 0.001
	Quadratic	−1.773 × 10^−5^	6.392 × 10^−6^	1905	−2.775	0.006
Irritation	Intercept	2.114 × 10^−0^	3.715 × 10^−2^	1905	56.892	< 0.001
	Linear	4.933 × 10^−3^	4.273 × 10^−4^	1905	11.544	< 0.001
	Quadratic	4.605 × 10^−5^	7.271 × 10^−6^	1905	6.333	< 0.001

DV, dependent variable; SE, standard error of the estimate; df, degrees of freedom; t, t-value; p, significance level.

The extrema of the parabolae are indicated by arrows: the quadratic models predict maximum *Valence* ratings for −60.3%, maximum *Entrainment* for −68.1%, maximum *Enjoyment* for −56.7%, and minimum *Irritation* for −53.7% *Signed-*Δ*-Magnitude*. All models predict the “point of greatest groove” at approximately −60% *Signed-*Δ*-Magnitude*, i.e., roughly halfway between the stimuli with fully quantized timing and the stimuli with original timing.

### 3.2. *Signed*-Δ-*Magnitude* × *Expertise* interaction

**Figure 4:**
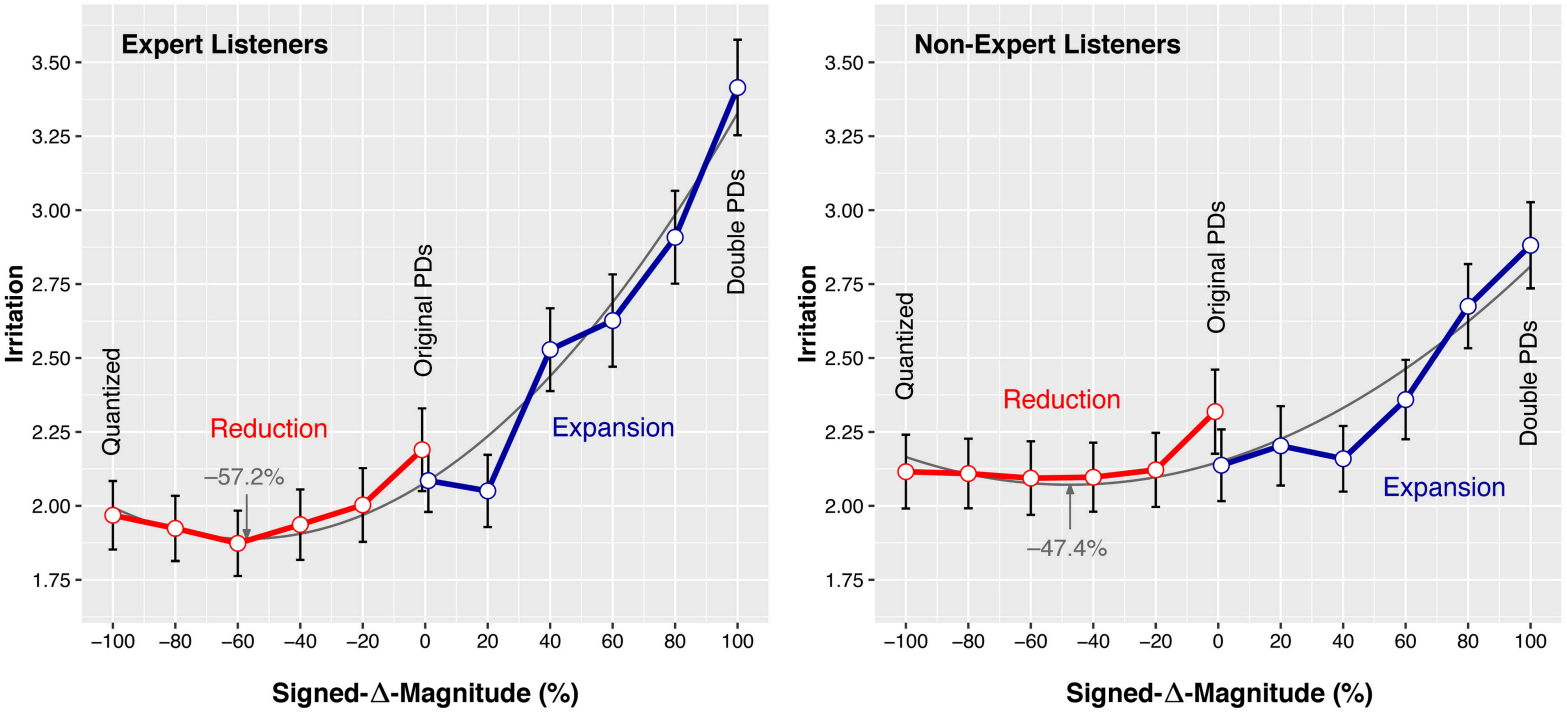
**Mean ***Irritation*** (***EAG***) ratings by Expert and Non-Expert listeners for each level of ***Signed***-Δ-***Magnitude*****. Quadratic polynomial lines of best fit are given in the background, minima are indicated by arrows. Error bars represent the standard error of the mean.

The *Signed-*Δ*-Magnitude* × *Expertise* interaction was significant for *Irritation* (Table 4). The timing manipulations had a significant impact on the *Irritation* ratings of both *Expertise* groups. But the effect on Experts [*F*_(11, 858)_ = 20.099, *p* < 0.001, η^2^ = 0.205] was considerably larger than the effect on Non-Experts [*F*_(11, 869)_ = 6.525, *p* < 0.001, η^2^ = 0.076]. Figure 4 presents the mean *Irritation* responses for the two groups. The plots show a similar general response pattern for Experts and Non-Experts: *Irritation* ratings are low for all negatively *Signed-*Δ*-Magnitude* levels, they increase for the higher positive levels of *Signed-*Δ*-Magnitude*. Expert listeners' responses show this pattern more distinctly than the Non-Expert listeners' responses.

Non-Experts reacted to exaggerated microtiming with seemingly little sensitivity: Tukey HSD pairwise comparisons (Table 7) show that the microtiming pattern needed to be exaggerated by +80% for Non-Expert listeners to react with significantly higher *Irritation* compared to any of the lower levels of *Signed-*Δ*-Magnitude*. The timing manipulations in the range from −100% to +60% did not significantly affect the *Irritation* of Non-Experts. The largest effect in Non-Experts was measured between the −40% and the +100% levels of *Signed-*Δ*-Magnitude* (*p* < 0.001, *d* = 0.684).

**Table 7:** *****Post-hoc*** pairwise comparisons (Tukey HSD) of mean ***Irritation*** ratings for levels of ***Signed-***Δ***-Magnitude***, separated by ***Expertise*** groups**.

**Signed-**Δ**-magnitude**			**Expert listeners**		**Non-expert listeners**	
			***p***	***d***	***p***	***d***
−100%	⇄	+40%	0.013	0.505	0.999	–
		+60%	0.001	0.554	0.855	–
		+80%	< 0.001	0.788	0.005	0.480
		+100%	< 0.001	1.188	< 0.001	0.649
−80%	⇄	+40%	0.004	0.556	0.999	–
		+60%	< 0.001	0.602	0.833	–
		+80%	< 0.001	0.841	0.004	0.499
		+100%	< 0.001	1.246	< 0.001	0.671
−60%	⇄	+40%	0.001	0.601	0.999	–
		+60%	< 0.001	0.644	0.770	–
		+80%	< 0.001	0.882	0.002	0.500
		+100%	< 0.001	1.285	< 0.001	0.668
−40%	⇄	+40%	0.006	0.527	0.999	–
		+60%	< 0.001	0.575	0.784	–
		+80%	< 0.001	0.807	0.003	0.511
		+100%	< 0.001	1.203	< 0.001	0.684
−20%	⇄	+40%	0.028	0.458	0.999	–
		+60%	0.003	0.510	0.876	–
		+80%	< 0.001	0.738	0.005	0.474
		+100%	< 0.001	1.128	< 0.001	0.642
−0%	⇄	+80%	< 0.001	0.558	0.326	–
		+100%	< 0.001	0.934	0.004	0.449
+0%	⇄	+60%	0.020	0.469	0.919	–
		+80%	< 0.001	0.710	0.008	0.468
		+100%	< 0.001	1.122	< 0.001	0.639
+20%	⇄	+60%	0.009	0.475	0.994	–
		+80%	< 0.001	0.705	0.041	0.393
		+100%	< 0.001	1.099	< 0.001	0.557
+40%	⇄	+80%	0.339	–	0.014	0.466
		+100%	< 0.001	0.675	< 0.001	0.642
+60%	⇄	+100%	< 0.001	0.571	0.012	0.428
+80%	⇄	+100%	0.041	0.366	0.950	–

p: significance level; d: Cohen's d. Comparisons without significant effects in any of the Expertise groups are omitted.

Expert listeners reacted more sensitively to exaggerated microtiming than Non-Experts: the pairwise comparisons of Table 7 show that, at the +40% level, *Irritation* ratings were significantly higher than the ratings for several lower levels of *Signed-*Δ*-Magnitude*. The difference of the ratings between the neighboring +20% and +40% levels was near-significant (*p* = 0.073). We can summarize that the timing manipulations did not significantly affect the *Irritation* of Experts in a range between −100% and +20% but increased strongly above +20%. The largest effect in Experts was measured between the −60% and the +100% levels of *Signed-*Δ*-Magnitude* (*p* < 0.001, *d* = 1.285).

Quadratic regression models (Table 8) support the above observations: The relative size of the quadratic coefficients implies that Experts reacted more strongly to changes in *Signed-*Δ*-Magnitude* than Non-Experts: the parabola summarizing the Experts' responses in Figure 4 is narrow compared to the wide parabola modeling the Non-Experts' responses. Experts' higher sensitivity to exaggerated microtiming is emphasized by the location of the “points of least irritation:” for Experts, this point is slightly more to the negative side of *Signed-*Δ*-Magnitude* (−57.2%), compared to Non-Experts (−47.4%).

**Table 8:** *****Post-hoc*** quadratic polynomial regression models for the relationship between ***Signed-***Δ***-Magnitude*** (predictor) and ***Irritation*** (response), separated by ***Expertise*** groups**.

**Expertise**	**Coefficient**	**Estimate**	**SE**	***df***	***t***	***p***
Expert	Intercept	2.079 × 10^−0^	5.292 × 10^−2^	945	39.279	< 0.001
	Linear	6.663 × 10^−3^	6.087 × 10^−4^	945	10.946	< 0.001
	Quadratic	5.827 × 10^−5^	1.036 × 10^−5^	945	5.625	< 0.001
Non-expert	Intercept	2.148 × 10^−0^	5.172 × 10^−2^	957	41.535	< 0.001
	Linear	3.224 × 10^−3^	5.948 × 10^−4^	957	5.421	< 0.001
	Quadratic	3.398 × 10^−5^	1.012 × 10^−6^	957	3.357	0.001

SE, standard error of the estimate; df, degrees of freedom; t, t-value; p, significance level.

### 3.3. Correlations between dependent variables

In this study, four dependent variables (*Valence, Entrainment, Enjoyment*, and *Irritation*) were associated with the timing manipulations. These variables showed a distinct pattern of correlations: *Valence, Entrainment*, and *Enjoyment* were mutually positively correlated, the strongest correlation was observed between the *EAG*'s *Entrainment* and *Enjoyment* variables (Table 9). *Irritation* was negatively correlated with the three other variables. This pattern of correlations among the *EAG* scales was expected by design.

**Table 9:** **Pearson product-moment correlations between dependent variables**.

	**Valence**	**Entrainment**	**Enjoyment**	**Irritation**
Entrainment	0.538			
Enjoyment	0.635	0.672		
Irritation	−0.607	−0.414	−0.499	
Movement	–	0.148	–	–

Movement refers to the Mean Periodic Head Movement variable in Kilchenmann and Senn (2015). Only significant correlations are reported, α = 0.05.

*Beat-Related Periodic Head Movement* in participants (*Movement*) was the dependent variable of Kilchenmann and Senn (2015). As Table 9 shows, *Movement* was positively, but weakly correlated with the *EAG*'s *Entrainment* scale.

### 3.4. Personality, affective state, and gender

The participants' mean *NEO-ffi* and *PANAS* scores are presented in Table 10. On average, the Experts scored higher on *Neuroticism* than the Non-Experts [*t*_(150)_ = 2.912, *p* = 0.004, *d* = 0.462]. With respect to the other personality factors, the two *Expertise* groups did not differ significantly. The table also presents *NEO-ffi* scores from a representative German sample (*n* = 1908) as a reference (Körner et al., 2002). The present study's sample of young adults scored high on *Extraversion* and *Openness*, which agrees with observations on this age stratum reported by Körner et al. (2002). We found no significant correlations between personality measures and *EAG* groove ratings or head *Movement* intensity.

**Table 10:** **Mean ***NEO-ffi*** and ***PANAS*** scores**.

	**Experts**	**Non-experts**	**Reference**
***NEO-ffi***			
Neuroticism	1.80 (0.66)	1.53 (0.54)	1.62 (0.62)
Extraversion	2.47 (0.48)	2.55 (0.52)	2.20 (0.50)
Openness	2.78 (0.49)	2.72 (0.52)	2.05 (0.46)
Agreeableness	2.77 (0.49)	2.80 (0.47)	2.54 (0.47)
Conscientiousness	2.61 (0.59)	2.72 (0.54)	2.71 (0.55)
***PANAS***			
Positive affects	35.59 (5.29)	36.76 (5.56)	32.85 (5.52)
Negative affects	18.81 (6.03)	17.70 (4.57)	18.36 (5.64)

The NEO-ffi reference data was reported by Körner et al. (2002), the PANAS reference data by Krohne et al. (1996). Standard deviations are given in parentheses.

The two *Expertise* groups scored similarly on both positive and negative affects of the *PANAS*. As a reference, Table 10 (bottom) presents scores from a German sample (*n* = 480) reported by Krohne et al. (1996). In comparison, this study's participants scored high on positive affects, but no difference was measured for negative affects. Affect measurements were not correlated with *EAG* ratings, *SAM* ratings or with head *Movement*.

We can conclude that personality traits and habitual affective states did not differ between *Expertise* groups, they were similar to those measured in reference samples, and they were not correlated to the dependent variables of the study. Finally, *EAG* and *SAM* ratings did not differ significantly between male and female participants.

## 4. Discussion

In this study, we systematically manipulated the magnitude of microtiming deviations in swing and funk rhythm section performances. Our goal was to assess how the manipulations affect the groove ratings of expert and non-expert listeners. We hypothesized that the microtiming patterns of the originally recorded performances would receive high groove ratings in comparison to microtiming patterns that were either reduced or expanded in magnitude. We further hypothesized that expert listeners would be more sensitive to microtiming manipulations than non-experts and that the funk stimuli would receive higher groove ratings than the swing stimuli.

As predicted, the stimuli with the originally performed microtiming patterns received high groove ratings. However, ratings did not decline symmetrically in both directions as timing deviations were reduced or expanded along the *Signed-*Δ*-Magnitude* variable (Figure 3). Rather, ratings were generally high for all stimuli with reduced microtiming, whereas ratings for the stimuli with expanded microtiming decreased as timing deviations became larger. We observe a “high groove zone” that extends from the quantized stimuli to the stimuli with the originally performed timing and slightly beyond, depending on the response variable. This confirms one major aspect of *PD theory*, namely that expert performer microtiming is rated high on groove. But it also corroborates the findings by Frühauf et al. (2013) and Davies et al. (2013) that quantized stimuli receive high groove ratings, thus confirming the *exactitude hypothesis*. The original performances appear to maximize microtiming deviations without compromising the groove experience: *Irritation* ratings were low for 0% *Signed-*Δ*-Magnitude*, but started to increase as microtiming deviations were expanded beyond the original magnitude.

These findings contrast with results from Kilchenmann and Senn (2015) in two particular instances: In the earlier study, the quantized stimuli were associated with low mean periodic head movement in expert listeners. In the present study, however, the completely quantized stimuli received high ratings on the *EAG*'s *Entrainment* scale. Conversely, in the 2015 study, the experts showed increased periodic head movement in response to stimuli with exaggerated microtiming. In the present study, those stimuli obtained low *Entrainment* ratings. In both cases, the self-reported experience of entrainment did not match the participants' actual bodily entrainment behavior. Across the two studies, *Periodic Head Movement Intensity* (Kilchenmann and Senn, 2015) was positively, but only weakly correlated with *Entrainment* [*r* = 0.148, *t*_(1906)_ = 6.535, *p* < 0.001, see also Table 9].

This means that listeners did not always move along with the music, when they reported an urge to move, and they did not always report an urge, when in fact they did move along with the music. For exaggerated timing, the observations offer some support to an explanation proposed in Kilchenmann and Senn (2015). There, we speculated that listeners potentially entrain to music for other reasons than groove. For example, they might clarify a rhythmically ambiguous situation by externalizing their sense of the beat through body movement. This follows findings by Phillips-Silver and Trainor (2007) and Manning and Schutz (2013) which suggest that moving with music can modify rhythm perception, compared to passive listening. The present paper's *EAG* ratings show that expert listeners rated stimuli with exaggerated microtiming to be low on groove, even though they displayed inreased entrainment to these stimuli, as reported in the earlier study. So, the magnitude of entrained body movement is not necessarily associated with groove ratings. It seems that experiencing the enjoyable groove urge is not the only reason why people entrain to music.

For the quantized timing, the contrast between weak head movement in expert listeners and high *Entrainment* ratings is puzzling: listeners experienced the urge to move, but they did not act on it. Why would they hold back? It seems that the expert listeners thought the quantized music inspired them to movement, when in fact it did not. Followers of *PD theory* might claim that the quantized stimuli lacked PDs and therefore did not have the power “to make us dance, make us want to participate” (Keil, 1995). But if this is the case, why would the highly trained and competent expert listeners not notice this lack of power and rate the quantized stimuli low on groove? At the time being, we do not have a plausible explanation for this discrepancy.

In one instance, expert listeners' periodic head movements (see Figure 4A in Kilchenmann and Senn, 2015) paralleled this study's groove ratings (see Figure 3 in the present paper): at the −60% *Signed-*Δ*-Magnitude* level, both head movements and groove ratings peaked at least nominally. So, expert listeners reported a strong subjective groove experience while listening to music with tight, but non-zero microtiming; and they accompanied this with intense entrained head movements. The *post-hoc* quadratic regression models presented in the Results section seem to confirm this observation: firstly, they suggest that the groove ratings can successfully be modeled as a curvilinear function of timing deviation sizes, secondly, all four models predict their “points of best groove” in the proximity of −60%.

In contrast, the high groove ratings for the quantized music were not accompanied by a strong bodily entrainment reaction. On the −60% level, the two measurements for groove agree (high ratings, intense bodily entrainment), while they disagree for the quantized stimuli (high ratings, but little bodily entrainment). This offers some weak evidence against the *exactitude hypothesis*: if we consider groove to involve a positive emotional reaction strictly coupled with bodily entrainment, then this study's quantized stimuli may not be strongly associated high groove. However, the case against the *exactitude hypothesis* is far from being conclusive.

Findings support our second hypothesis that experts would be more sensitive to microtiming manipulations than non-experts. Both experts and non-experts showed a similar general irritation response pattern to the microtiming manipulations: small PDs were associated with low irritation, and exaggerating the magnitude of the PDs beyond the originally performed magnitude eventually irritated the listeners. But expert listeners reacted more strongly to the manipulations, compared to the non-expert listeners. Also, experts were more sensitive to the magnitude of the timing manipulation: An increase of PD magnitudes by +40% was enough to trigger irritated responses by the experts, whereas non-experts' irritation increased significantly only when PD magnitudes were exaggerated by +80% or more. This resonates with results from Kilchenmann and Senn (2015) who reported measureable effects of the timing manipulation on the experts' body movement behavior, while not registering significant effects on non-experts. This is also in line with the findings of Davies et al. (2013) who observed that expert listeners used the range of the rating scales more widely than the non-experts.

The expert group's sensitivity to microtiming deviations is not surprising: we expect music experts to have a refined perception of timing nuance, due to their experience and training. However, this dependency on expertise casts a doubt on the claim of *PD theory* that microtiming generally enhances the groove experience. Non-experts seem to react less to microtiming phenomena than the experts. So, if PDs turn out to have an influence on the groove experience, musical expertise might be an important mediator that determines whether this influence is felt or not. Given that musical experts were strongly involved in the development of *PD theory*, the claims of this theory might predominantly reflect their expert perspective on music. The PD effect might turn out to be a treat for the musical elite.

Apart from expertise, we did not find any other person-related effects on groove reactions. Affective disposition (*PANAS*) or personality dimensions (*NEO-ffi*) were not associated with groove ratings or head movement.

Our third hypothesis postulated that the funk clips would receive higher groove ratings than the swing clips, because funk is a musical genre traditionally associated with groove. The data does not support this claim: the style variable in our study was not associated with any of the dependent variables. This is in line with our previous findings that *Style* did not influence head movement (Kilchenmann and Senn, 2015). Note that participants either listened to swing or to funk stimuli, but no participant heard stimuli of both styles. So, no direct comparison between stimuli from different styles took place. We can probably expect effects of music preference and taste to become relevant, when participants assess stimuli from different styles in the same experiment.

On the surface, the non-result concerning *Style* is unremarkable: listeners reacted similarly to the timing manipulations in swing and funk. However, if we consider the differences between the originally performed microtiming patterns and magnitudes that were the basis of these manipulations, the result is quite interesting: listeners reacted similarly to equivalent proportional timing manipulations in both styles, even though the absolute microtiming magnitudes were different for each style.

How can we compare the microtiming magnitudes of the swing and funk performances? Previous studies have shown that the just-noticeable difference of timing deviations in isochronous auditory sequences depends on tempo (Friberg and Sundberg, 1995; Ehrlé and Samson, 2005) and that the magnitudes of timing deviations in simple sensori-motor tasks are positively associated with the width of the inter-onset-intervals (Madison, 2001; Repp, 2005). Taking this into account, we introduce the *tempo-adjusted standard timing deviation*, *s*_Δ*t*(*B*)_, as a summary statistic for quantifying the magnitude of microtiming phenomena in performed music. It is calculated as follows:

$$\begin{array}{l} s_{\Delta t\text{(B)}}\;=\;\frac{\text{bpm}}{60}\sqrt{\frac{1}{n}\sum_{i=1}^{n}{\left(t_{i}-{\hat{t}}_{i}\right)}^{2}}, \end{array}$$

where *t*_*i*_ is the absolute time of the *i*th event onset in seconds; ${\hat{t}}_{i}$ is the absolute time of that onset's metronomic grid position (or quantized position) in seconds; *n* is the overall number of events in the music clip, and *bpm* is the tempo of the music in beats per minute. The tempo-adjusted standard timing deviation is given as a fraction of the metronomic beat duration.

The timing deviations found in the two originally performed and recorded clips can be inspected in Figures 1, 2. The standard timing deviation measured in the funk clip is *s*_Δ*t*(*B*)_ = 0.026 beats; in the swing clip, it amounts to *s*_Δ*t*(*B*)_ = 0.068 beats. The large *s*_Δ*t*(*B*)_ difference between funk and swing depends on the absolute deviations (in ms), which were larger in swing than in funk, but also on the tempo difference (150 bpm in swing vs. 100 bpm in funk).

The large timing deviations in swing and the much smaller timing deviations in funk resulted in similar groove ratings when scaled according to the same rules. So, both the performing musicians and the listeners appear to agree on how much microtiming is acceptable in either style. One remarkable aspect of this result consists in the fact that this observation not only concerns the expert group among the participants. The non-experts react similarly to timing manipulations in the two styles, they were simply less sensitive to exaggerated timing deviations. Overall, there seems to be at least an implicit style-dependent notion of adequate vs. exaggerated microtiming in the analyzed population, regardless of expertise.

The standard timing deviation also allows us to draw a comparison between this study's results and the findings of Frühauf et al. (2013). In their study, the timing of a generic rock drum pattern at tempo 120 bpm was manipulated by displacing two events per measure while leaving all other events quantized. The maximum displacement was 25 ms; and in all permutations (early vs. late, snare drum vs. bass drum), the maximum displacement led to significantly lowered groove ratings, when compared to the quantized version. At maximum displacement, the microtiming patterns had a standard timing deviation of *s*_Δ*t*(*B*)_ = 0.020 beats, which is smaller than the *s*_Δ*t*(*B*)_ = 0.026 beats measured for the originally performed funk example of the present study (and much smaller than the *s*_Δ*t*(*B*)_ = 0.068 of the originally performed swing example). We can conclude that the ratings reported by Frühauf et al. (2013) were sensitive to relatively small timing deviations, whereas the ratings in the present study were more robust: at the original microtiming magnitude, this study's stimuli were rated high on groove.

Comparisons across studies must always be carried out with caution. The two studies differ in so many respects (instrumentation, musical content, experimental setup, measurement methods, etc.) that no definite conclusions can be drawn. However, we can at least formulate a new hypothesis: we suspect that the patterning of the microtiming deviations makes a relevant difference. Frühauf et al. (2013) created a perfectly quantized pattern and displaced a few of the events. Research on vigilance and attention has shown that irregular signals against a regular background have a high potential of being detected (Scerbo et al., 1986; Bregman, 1999; Parasuraman, 2000; Helton et al., 2005; Dalton et al., 2007; Winkler et al., 2009), regardless of the mode of perception (visual or auditory). Hypothetically, the artificially manipulated events in Frühauf et al. (2013) stood out against the background of the quantized pattern and led to lower groove ratings. In contrast, the microtiming patterns in the present study were created in an interactive performance situation. The distribution of microtiming deviations was more varied, and almost every event showed some temporal distance from the metronomic grid. Under these circumstances, no potentially irritating contrast between a quantized background and an out-of-sync foreground can emerge. Future studies might investigate microtiming patterns in depth (following the example of Hellmer and Madison, 2015), and test the effects of these patterns on the groove experience.

A final note on methodology: for Kilchenmann and Senn (2015) and for the present study, two different methods of assessing groove have been applied within the same experiment: motion tracking and questionnaires. This allowed to relate the results and put them into perspective. Both approaches have their strengths and weaknesses: the direct measurement of bodily movement through motion tracking has the advantage of registering a spontaneous and largely unreflected entrainment reaction that is thought to be closely associated with the groove phenomenon. Its drawbacks include that the effects seemed to be considerably smaller than the effects measured using questionnaires, and that the movement behavior was not qualified by the participants: we cannot distinguish between entrained behavior as a result of groove experience and entrainment due to other reasons. Questionnaires like the *EAG* allow to ask very specific questions, and the measured effects seem to be quite strong. On the downside, filling a questionnaire with several scales and several items per scale is time-consuming: the 20-item *EAG* was rather too long for application in a listening experiment. The laborious task tested the patience of many participants, which in turn may influence the groove experience negatively. Hence, it is certainly beneficial to shorten the questionnaire, preferably without compromising reliability. A first step is reducing the *EAG* to the 13 items that have been used in the present analysis. In due course, an English version of the questionnaire will be prepared and made available to the scientific community.

A central methodological problem lies in the disagreement between results derived from the two approaches: participants' bodily entrainment behavior and their subjective groove ratings (particularly on the *Entrainment* scale) rarely agreed with each other. If both methods assess the intensity of the same underlying experience, then the results should confirm one another. This, however, was not always the case. It seems that the groove experience is not that easy to measure. It will take considerable effort to develop reliable methods to assess a listener's groove experience and bodily entrainment.

## 5. Conclusions

Past research (Davies et al., 2013; Frühauf et al., 2013) has found that microtiming influences the groove phenomenon. The findings of the present study allow us to give a more specific answer to the question whether microtiming supports (*PD theory*) or weakens (*exactitude hypothesis*) the groove experience in listeners. Both perfectly quantized stimuli and stimuli with PD patterns arising during a competent performance obtained similarly high groove ratings. Hence, if we interpret the theories in their strict mutually exclusive sense, neither is fully supported by the data: PDs are not mandatory for music to groove (as *PD theory* suggests), and the groove experience is not necessarily damaged when music is not perfectly quantized (as claimed by the *exactitude hypothesis*).

Our findings confirm previous results that exaggerated microtiming deviations diminish groove. But whether listeners consider microtiming magnitudes to be adequate or exaggerated seems to depend on musical genre and on the musical expertise of the listener. We suspect that the patterning of microtiming deviations is relevant, and we propose to study this aspect further in the future. From a more general perspective, it is unclear how much microtiming matters in the context of other features of the music.

The application of different measuring tools in this study and in Kilchenmann and Senn (2015) led to conflicting results, which indicates that the groove concept has not yet been fully understood. Future research must further clarify the psychological construct of groove and improve the measuring instruments. The scope of the investigation must also be widened beyond microtiming: some potentially relevant aspects (e.g., syncopation, beat salience) have been addressed by previous research, but many other aspects still await study. Potentially relevant aspects may include musical structure (rhythmic patterns, repetition, tempo), music's presentation or diffusion (loudness, sound quality, frequency spectrum), the situation in which it is consumed (concert, work-out, dance party, individual listening), and the person of the listener (taste, personal listening history, mood).

The groove experience appears to be a formidably complex and multilayered phenomenon. Given its towering relevance for music appreciation in society, understanding how it works may well be one of the most important tasks in music psychology today.

## Author contributions

Conceived and designed the experiment: OS, LK, RV, CB. Designed the *EAG* questionnaire: RV, LK, CB, OS. Validated the *EAG* questionnaire: RV. Performed the experiment: LK. Analyzed the data: OS. Wrote the paper: OS, LK, RV, CB.

## Funding

This research was part of the project “The Relevance of Participatory Discrepancies for the Perception of Groove in Jazz and Funk,” supported by the Swiss National Science Foundation (grant 100012L 137794 to OS) and the Deutsche Forschungsgemeinschaft (grant BU 2259/1-1 to CB).

### Conflict of interest statement

The authors declare that the research was conducted in the absence of any commercial or financial relationships that could be construed as a potential conflict of interest.

## Footnotes

^1^http://journal.frontiersin.org/article/10.3389/fpsyg.2015.01232/abstract
