# Supplementary material for: The Effect of Expert Performance Microtiming on Listeners' Experience of Groove in Swing or Funk Music
Source: Front Psychol. 2016 Oct 5;7:1487. doi: 10.3389/fpsyg.2016.01487 (PMC5050221; doi:10.3389/fpsyg.2016.01487)
Supplement: Supplementary file 1 [file Presentation1.PDF]

Anweisungen zum Hörexperiment

Im Folgenden werden Sie einige kurze musikalische Beispiele hören. Wir möchten nach jedem dieser Beispiele von Ihnen wissen, wie Sie sich hierbei subjektiv gefühlt haben bzw. welche Gefühle dieses Beispiel bei Ihnen ausgelöst hat.

Um dieses zu Erfahren folgen im Anschluss eines jeden Beispiels zwei kurze Fragebögen, die Sie bitte schnell und ohne zu zögern beantworten sollen. Hierbei gibt es kein „Richtig" oder „Falsch", sondern nur Ihr persönliches Gefühl ist für uns von Bedeutung. Versuchen Sie bitte jegliches langes Nachdenken und Abwägen zu vermeiden, da für uns nur ihre ganz spontane Reaktion von Bedeutung ist.

Nach Beendigung des ersten musikalischen Beispiels folgt zunächst eine kurze Einschätzung ihrer emotionalen Befindlichkeit, die durch das Beispiel bei Ihnen ausgelöst wurde. Es folgen dann ein paar Fragen zur Einschätzung des Beispiels.

Bitte achten Sie darauf keine Frage auszulassen!

EAG Fragebogen

	trifft gar nicht zu				trifft voll zu
Ich empfand das Beispiel als groovy	☐	☐	☐	☐	☐
Das Beispiel animierte mich zum mitwippen	☐	☐	☐	☐	☐
Ich Hatte das Gefühl, dass irgendetwas störend wirkt	☐	☐	☐	☐	☐
Das Beispiel strahlte für mich eine gewisse Lockerheit aus	☐	☐	☐	☐	☐
Das Beispiel animierte mich zum Mitklatschen oder Schnippen	☐	☐	☐	☐	☐
Das Beispiel machte mich eher nervös und unruhig	☐	☐	☐	☐	☐
Ich empfand das Beispiel als anregend	☐	☐	☐	☐	☐
Irgendwie wirkte das Beispiel bremsend und/oder merkwürdig auf mich	☐	☐	☐	☐	☐
Ich empfand das Beispiel als frisch	☐	☐	☐	☐	☐
Ich konnte das automatische Mitticken meiner „inneren Uhr“ fühlen	☐	☐	☐	☐	☐
Das Beispiel hinterließ den Eindruck einer gewissen Holprigkeit, die mir eher unangenehm war.	☐	☐	☐	☐	☐
Das Beispiel war für mich sehr kraftvoll	☐	☐	☐	☐	☐
Ich hatte das Gefühl, dass ich mit meinem Fuß gerne Mitklopfen würde	☐	☐	☐	☐	☐
Ich empfand das Beispiel als eher langweilig	☐	☐	☐	☐	☐
Bei diesem Beispiel hätte mich interessiert, wie es weitergeht	☐	☐	☐	☐	☐
Ich hatte das Gefühl, dass sich mein Kopf zum Rhythmus mitbewegt	☐	☐	☐	☐	☐
Irgendetwas war mit dem Beispiel nicht in Ordnung und ich hatte ein merkwürdiges Gefühl	☐	☐	☐	☐	☐
Ich konnte den „Swing“ bzw. “Groove“ im Beispiel spüren	☐	☐	☐	☐	☐
Das Beispiel animierte mich dazu, in meinem Kopf eine Melodie (Töne) mit hinzuzufügen	☐	☐	☐	☐	☐
Ich hatte das Gefühl, dass die Instrumente sehr gut miteinander harmonieren	☐	☐	☐	☐	☐
